# Mapping autoantigen epitopes: molecular insights into autoantibody-associated disorders of the nervous system

**DOI:** 10.1186/s12974-016-0678-4

**Published:** 2016-08-30

**Authors:** Nese Sinmaz, Tina Nguyen, Fiona Tea, Russell C. Dale, Fabienne Brilot

**Affiliations:** 1 Brain Autoimmunity Group, Institute for Neuroscience and Muscle Research, The Kids Research Institute at the Children’s Hospital at Westmead, University of Sydney, Locked Bag 4001, Westmead, NSW 2145 Australia; 2 Discipline of Child and Adolescent Health, Sydney Medical School, University of Sydney, Sydney, Australia

**Keywords:** CNS disorders, PNS disorders, Autoantibody, Epitope mapping, Epitope spreading

## Abstract

**Background:**

Our knowledge of autoantibody-associated diseases of the central (CNS) and peripheral (PNS) nervous systems has expanded greatly over the recent years. A number of extracellular and intracellular autoantigens have been identified, and there is no doubt that this field will continue to expand as more autoantigens are discovered as a result of improved clinical awareness and methodological practice. In recent years, interest has shifted to uncover the target epitopes of these autoantibodies.

**Main body:**

The purpose of this review is to discuss the mapping of the epitope targets of autoantibodies in CNS and PNS antibody-mediated disorders, such as *N*-methyl-D-aspartate receptor (NMDAR), α-amino-3-hydroxy-5-methyl-4-isoxazolepropionic acid receptor (AMPAR), leucine-rich glioma-inactivated protein 1 (Lgi1), contactin-associated protein-like 2 (Caspr2), myelin oligodendrocyte glycoprotein (MOG), aquaporin-4 (AQP4), 65 kDa glutamic acid decarboxylase (GAD65), acetylcholine receptor (AChR), muscle-specific kinase (MuSK), voltage-gated calcium channel (VGCC), neurofascin (NF), and contactin. We also address the methods used to analyze these epitopes, the relevance of their determination, and how this knowledge can inform studies on autoantibody pathogenicity. Furthermore, we discuss triggers of autoimmunity, such as molecular mimicry, ectopic antigen expression, epitope spreading, and potential mechanisms for the rising number of double autoantibody-positive patients.

**Conclusions:**

Molecular insights into specificity and role of autoantibodies will likely improve diagnosis and treatment of CNS and PNS neuroimmune diseases.

## Background

Autoantibodies can be associated with diseases that affect the central nervous system (CNS) or peripheral nervous system (PNS). The associated autoantibodies can be directed against intracellular or extracellular autoantigens [1–3], and the clinical phenotype of patients is diverse, encompassing movement disorders and neuropsychiatric disorders [4, 5]. Antibodies targeting intracellular antigens are not considered pathogenic but are sometimes believed to be biomarkers of an underlying tumor [6]. Conversely, antibodies targeting extracellular domains of neuronal cell surface receptors or synaptic proteins, sometimes associated with a tumor, are generally considered to be pathogenic and can modulate receptor function or decrease receptor density at the cell surface [7–9]. Autoantigens found in CNS antibody-mediated disorders include *N*-methyl-D-aspartate receptor (NMDAR) [10], α-amino-3-hydroxy-5-methyl-4-isoxazolepropionic acid receptor (AMPAR) [11], glycine receptor (GlyR) [12], components of the voltage-gated potassium channel (VGKC) complex, including leucine-rich glioma-inactivated protein 1 (Lgi1) and contactin-associated protein-like 2 (Caspr2) [13–15], γ-aminobutyric acid receptor-B (GABA_B_R) [16], γ-aminobutyric acid receptor-A (GABA_A_R) [17], metabotropic glutamate receptor 5 (mGluR5) [18], dipeptidyl-peptidase-like protein-6 (DPPX) [19], dopamine-2 receptor (D2R) [20], myelin oligodendrocyte glycoprotein (MOG) [21–23], aquaporin-4 (AQP4) [24], 65 kDa glutamic acid decarboxylase (GAD65) [25], neurofascin (NF) [26], and contactin [27]. Furthermore, a number of different autoantigens have been discovered in neuromuscular junction antibody-mediated disorders. These include acetylcholine receptor (AChR) [28], muscle-specific kinase (MuSK) [29], lipoprotein receptor-related protein 4 (Lrp4) [30], all associated with myasthenia gravis (MG), and voltage-gated calcium channel (VGCC) associated with PNS Lambert-Eaton myasthenic syndrome (LEMS) [31].

The discovery of antibody-associated brain disorders has helped to identify patients amenable to immunotherapies, leading to more rapid recovery and improved patient outcome. Recently, research has focused on the discovery of autoantibody epitope(s) to understand the mechanism of autoantibody pathogenicity. Herein, we review reported target epitope(s) for known antibody-mediated disorders of the nervous system, address the relevance of these studies, explore the notion of epitope spreading, and discuss the issue of double autoantibody-positive patients.

## Epitope mapping of antibody-mediated brain disorders

### Methods to map autoantibody epitopes

An important factor to consider in the identification of autoantigens and their epitope targets is the methodology that is used in the experimental setting. Current practices include subcloning and mutating the antigen of interest, either by deleting entire regions or making point mutations. Autoantibody epitope specificity is usually investigated using a cell-based assay followed by fluorescent microscopy or flow cytometry analysis [9]. However, western blots [32], radioimmunoassays [33], and ELISAs [34] have also been used to determine antigenic epitopes. Fundamentally, pathogenic autoantibodies recognize cell surface antigens in their native conformation. As such, it is important to remember that the type of method utilized will therefore determine whether the antigenic epitope remains in its native three-dimensional conformation or is converted to a denatured or linear configuration. Indeed, the use of a live cell-based assay, ELISA, or western blot will influence protein conformation, and hence results, interpretation, and conclusions. Furthermore, the live or fixed/permeabilized status of cells used in cell-based assays is critical as the latter also affects conformation and has led to high prevalence of autoantibodies in healthy donors (recently reviewed in [9]). An overview of the methodology used in mapping the epitopes is given in Table 1.

**Table 1:** Epitope target(s) of CNS and PNS human autoantibodies

	CNS/PNS disorder	Antigen	Target: extra/intra	Main epitope	Other epitopes	Number of N-glycosylation sites	Sample type	Methodology	Reference
CNS	Anti-NMDAR encephalitis	Neuronal NMDAR	Extra	GluN1/NR1 subunit (ATD, N368/G369)	–	10 (N368^a^)	CSF	Subcloning/ICC	Gleichman et al. [35]
	SLE		Extra	NR2A/2B subunit (LBD/S1, aa283–287)		6	Sera/CSF	Phage display peptide library	DeGiorgio et al. [67].
	Anti-AMPAR encephalitis	Neuronal AMPAR	Extra	GluR1 subunit (Bottom of ATD)	S1 domain	6	Sera/CSF	Subcloning/ICC, Fusion protein-based western blot	Gleichman et al. [32]
	Autoimmune encephalitis	Neuronal Caspr2	Extra	Disc domain	Lam1, Lam2, Egf1 domains	12	Sera	Subcloning/ICC, western blot	Olsen et al. [37]
			Extra	Disc, Lam1 domains	–		Sera/CSF	Flow cytometry cell-based assay	Pinatel et al. [38]
	Encephalitis/SPS	Neuronal GAD65	Intra	Catalytic region (aa221-444)	C-terminal domain	0	Sera	Immunoprecipitation assay	Fouka et al. [48]
			Intra	aa4-22, aa308-365, aa451-585	–		Sera	Competition assay	Raju et al. [47]
	NMOSD/BON/ADEM/CRION	Oligodendrocytes - MOG	Extra	β strand/CC’-loop (P42)	FG-loop (H103/S104)	1 (N60^a^)	Sera	Subcloning/cell-bound reactivity assay	Mayer et al. [56]
	NMO	Astrocytes - AQP4 isoform M23	Extra	Loop A (aa61-64)	Loop C (aa146-150) Loop E (W227)	2	Sera	Subcloning/ICC	Pisani et al. [62]
			Intra	aa1-22, Loop B (aa88-113), aa252-275	–		Sera	ELISA	Kampylafka et al. [61]
			Extra	Loop A (aa66-69)	Loop C (N153), Loop E (H230)		Sera/CSF	Cell-based assay	Tuller et al. [65]
PNS	MG	Muscle - AChR	Extra	α1 subunit (aa67-76)	–	1	Sera	Solid-phase radioimmunoassay	Tzartos et al. [33]
			Extra	α1 subunit (aa1-14, aa67-76)	–		Sera	Radioimmunoassay	Luo et al. [79]
			Extra	α1 subunit (N68, D71)	–		Sera	Peptide competition binding assay	Papadouli et al. [80] Wahlsten et al. [81]
			Intra	–	α1 subunit (aa373–380), β1 subunit (aa354–359)		Sera	Competition assay	Tzartos et al. [82]
	MG	Muscle - MuSK	Extra	Ig-like domain 1	Ig-like domain 2, Ig-like domain 3/ Fz-like domain	2	Sera	Radioimmunoprecipitation assay	McConville et al. [84]
			Extra	Ig-like domain 1, Ig-like domain 2	–		Sera	Radioimmunoprecipitation assay	Ohta et al. [85]
			Extra	Ig-like domain 1	Ig-like domain 2		Sera	Competition binding, ELISA, subcloning/ICC	Huijbers et al. [83]
			Extra	Ig-like domain 1	Ig-like domain 2, Fz-like domain		Sera	ELISA	Huijbers et al. [34]
	MG	Titin	Intra	Ig C2-like domain, Fn-like domain (MGT30)	–	0	Sera	cDNA expression library	Gautel et al. [88]
	LEMS	Neuronal P/Q-type VGCC	Extra	α1 subunit (linker region of S5-6 in repeated domains II, III, IV)	β1 subunit	1	Sera	Radioimmunoprecipitation assay	Parsons et al. [94]
			Extra	α1 subunit (linker region of domain IV)	α1 subunit (linker region of domain II)		Sera	Immunoprecipitation assay	Takamori et al. [95]
			Extra	α1 subunit (linker region of domain III)	–		Sera	Immunoblot	Iwasa et al. [96]
	NMT	Neuronal Caspr2	Extra	Disc domain	Lam1, Lam2, Egf1	12	Sera	Subcloning/ICC, Western blot	Olsen et al. [37]
			Extra	Disc, Lam1 domains	–		Sera/CSF	Flow cytometry cell-based assay	Pinatel et al. [38]
	NPSLE	Neuronal NMDAR	Extra	GluN1/NR1 (aa19-44, aa56-81)	–	10	Sera/CSF	ELISA	Ogawa et al. [72]
	CIDP, GBS, AMAN, AIPD	Neuronal NF186	Extra	Fn V domain, Mucin-like domain	–	8	Sera	Cell-based assay	Ng et al. [107]
		Glial NF155	Extra	Fn III domain, Fn IV domain					
	CIDP, GBS	Neuronal Contactin	Extra	Ig C2-like domain V^b^	–	9	Sera	Subcloning/ICC	Labasque et al. [108]

*AChR* acetylcholine receptor, *AIPD* acute inflammatory demyelinating polyradiculoneuropathy, *AMAN* acute motor axonal neuropathy, *AMPAR* α-amino-3-hydroxy-5-methyl-4-isoxazolepropionic acid receptor, *ADEM* acute disseminated encephalomyelitis, *AQP4* aquaporin-4, *BON* bilateral optic neuritis, *Caspr2* contactin-associated protein-like 2, *CIDP* chronic inflammatory demyelinating polyneuropathy, *CNS* central nervous system, *CRION* chronic relapsing inflammatory optic neuropathy, *CSF* cerebrospinal fluid, *DNA* deoxyribonucleic acid, *ELISA* enzyme-linked immunosorbent assay, *Fn* fibronectin-type III, *Fz* Frizzled, *GAD65* 65 kDa glutamic acid decarboxylase, *GBS* Guillain-Barre syndrome, *ICC* immunocytochemistry, *Ig* immunoglobulin, *LBD* ligand binding domain, *LEMS* Lambert-Eaton myasthenic Syndrome, *Lgi1* leucine-rich glioma-inactivated protein 1, *MG* myasthenia gravis, *MOG* myelin oligodendrocyte glycoprotein, *MS* multiple sclerosis, *MuSK* muscle-specific kinase, *NF* neurofascin, *NMDAR* *N*-methyl-D-aspartate receptor, *NMO* neuromyelitis optica, *NMOSD* neuromyelitis optica syndrome disorder, *NMT* acquired neuromyotonia, *NPSLE* neuropsychiatric systematic lupus erythematosus, *PNS* peripheral nervous system, *SPS* Stiff Person Syndrome, *T1D* type 1 Diabetes, *VGCC* voltage-gated calcium channel, *VGKC* voltage-gated potassium channel, - not applicable

^a^Potentially important glycosylation site involved in antibody binding and formation of epitope

^b^N-glycosylation sites N467/N473/N494 involved in antibody binding

## Autoimmune encephalitis

**Fig. 1:**
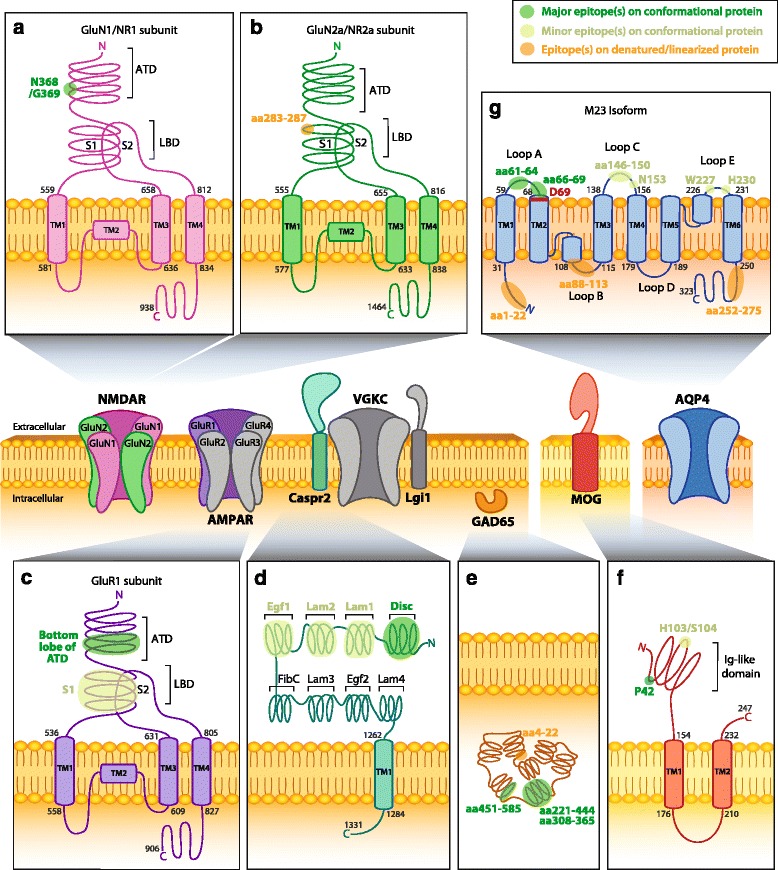
Epitopes of human CNS autoantibodies. Two subtypes of ionotropic glutamate receptors (iGluRs) are the *N*-methyl-D-aspartate receptor (NMDAR) and the α-amino-3-hydroxy-5-methyl-4-isoxazolepropionic acid receptor (AMPAR). Subunits of NMDAR (**a**, **b**) and AMPAR (**c**) contain an extracellular amino terminal domain (*ATD*) and a ligand-binding domain (*LBD*) formed by segment 1 (*S1*) and segment 2 (*S2*). Anti-NMDAR antibodies recognize amino acid (aa) N368 and G369 which reside on the bottom lobe in the ATD of the GluN1 subunit (**a**, Q05586) [35]. Anti-NMDAR antibodies also bind to an epitope at aa283-287 within S1 of the LBD in subunits GluN2a and GluN2b (**b**, Q12879; only GluN2A is shown) [67]. Anti-AMPAR antibodies are directed against an extracellular epitope within the bottom lobe in the ATD of the GluR1 subunit (**c**, P42261), but specific aa in the ATD have not been mapped [32]. **d** Contactin-associated protein 2 (Caspr2, Q9UHC6) consists of eight domains and forms part of the voltage-gated potassium channel (VGKC) complex with the leucine-rich glioma-inactivated protein 1 (Lgi1). Anti-Caspr2 antibodies recognize the extracellular N-terminal half (domains I–IV; discoidin (*Disc*), lamininG (*Lam*)*-1*, *Lam2*, and epidermal growth factor 1 (*Egf1*), respectively), but most commonly bind to an epitope within the Disc domain [37, 38]. **e** Anti-cytoplasmic enzyme glutamic acid decarboxylase 65 (GAD65) antibodies recognize an epitope at aa221-444 [48], aa451-585, and aa308-365 [47]. Anti-GAD65 antibodies have also been shown to bind to linearized protein at aa4-22 [47]. **f** Myelin oligodendrocyte glycoprotein (MOG, Q16653) is a myelin protein expressed on oligodendrocytes. Anti-MOG antibodies recognize a epitopes at P42 and at H103/S104 within the immunoglobulin (Ig)-like domain [56]. **g** Aquaporin-4 isoform M23 (AQP4-M23) is a water channel expressed on astrocytes. Anti-AQP4-M23 antibodies recognize epitopes within *loop C* (*aa146-150*) and *loop E* (*aa227-228*), but mostly in *loop A* (*aa61-64*) [62]. An additional study identified extracellular epitopes within *loop A* (*aa66-69*), *loop C* (*N153*), and *loop E* (*H230*) [65]. Reported intracellular epitopes include *aa1-22*, *aa88-113*, and *aa252-275* [61]. *D69* (*red*) is vital in maintaining the conformational structure of *loop A* [63]. Human AQP4-M23 sequence is derived from [210, 211]. Human protein topology, i.e., aa sequences and transmembrane domains (TM), is adapted from UniProt database and UniProt identifiers are shown between *brackets*. Diagrams do not depict protein crystal structure. *Green* highlights indicate major (*dark green*) and minor (*light green*) epitopes mapped by methods which retain the native in vivo conformational structure of proteins, such as cell-based assays. Orange highlights epitopes determined by methods that denature or linearized proteins, such as western blots and ELISAs (Table 1)

Anti-NMDAR encephalitis is the most commonly recognized autoimmune encephalitis (reviewed in [1]). It is associated with anti-NMDAR antibodies, has been reported in females and males, and has been observed in all age groups, from young children to the elderly [13]. Due to the severity of anti-NMDAR encephalitis, as well as its rising prevalence, several studies have strived to uncover the target epitope of anti-NMDAR antibodies and also to explore the mechanisms of anti-NMDAR antibody pathogenicity. NMDARs are composed of two GluN1 (also called NR1) and two GluN2/3 (also called NR2 and NR3) subunits that form a central ion channel (Fig. 1). Importance of the conservation of conformational antigen has been shown by the lack of immunoreactivity of anti-NMDAR antibody on immunoblots [10]. In 2012, the epitope of anti-NMDAR antibodies was mapped using a series of NMDAR mutants expressed in human embryonic kidney (HEK)293 cells that were incubated with CSF from patients with and without anti-NMDAR encephalitis [35]. The first step was to distinguish which subunit was involved in antibody binding. Anti-NMDAR antibody-positive CSF did not recognize the GluN2 variant of NMDAR by immunocytochemistry, but they did recognize GluN1 subunit and a splice variant of GluN1 [35]. GluN subunits are made up of two extracellular domains; the amino (N)-terminal domain (ATD) and the ligand-binding domain (LBD) consisting of segment 1 (S1) and segment 2 (S2), followed by three transmembrane-spanning domains (TM1, 3, 4), a transmembrane loop (TM2), and an intracellular carboxyl (C)-terminal domain (Fig. 1a). To determine whether it was the extracellular ATD or S1/S2 domains that were important for antibody binding, two different constructs were created. The first mutant included only the ATD domain and missed the S1/S2 domains, which resulted in intense patient antibody staining. The second construct excluded ATD and contained S1/S2 only, which abolished antibody binding [35]. These results suggested that the ATD of GluN1 is necessary for antibody recognition. Additional deletion constructs were created, including single-point mutations within the ATD, to define specific antibody binding. Among these, the most important mutant was N368D/G369I double mutant, which prevented patient antibodies from binding. Therefore, residues N368/G369 were found to be important targets of anti-NMDAR antibodies. As expected, control patients without anti-NMDAR antibodies did not stain for NMDAR mutants. Interestingly, the ATD of NMDAR includes seven putative N-linked glycosylation sites, one of which found at position N368. Analysis of N368Q mutant with patient samples showed reduced immunoreactivity but did not abolish antibody binding to the same extent as the double mutation of N368/G369 [35]. This result is interesting because it suggests that N-glycosylation sites are involved in creating the ideal epitope conformation. Pathogenic studies, discussed below, have also been carried out using this mutant.

Anti-AMPAR antibodies are detected in patients with anti-AMPAR encephalitis in a small number of described cases [11, 36]. AMPAR is an ionotropic glutamate receptor comprising of four subunits, GluR1-4 (Fig. 1). The extracellular domain contains ATD, LBD, three transmembrane domains, a transmembrane loop, and a C-terminal cytoplasmic tail. In 2014, the epitope of AMPAR was mapped [32] (Fig. 1c). Two different methods were employed to test antibody specificity in patient serum and CSF: immunocytochemistry following expression of mutated AMPAR in HEK293 cells and western blots using mutant AMPAR fused to thioredoxin tag expressed in bacteria. Anti-AMPAR antibodies were found to react to both GluR1 and GluR2 subunits of AMPAR. Deletion of S1 domain resulted in little to no immunoreactivity, and mutants lacking the bottom lobe of ATD prevented antibody binding. These results indicate that the main immunogenic targets of anti-AMPAR antibodies are located within the bottom lobe of ATD, but the precise residues were not mapped (Fig. 1c). In the case of AMPAR, although its tertiary structure is similar to the one of NMDAR and the AMPAR epitopes were located within the same glycosylation-prone region, the influence of glycosylation was not demonstrated. Anti-AMPAR antibodies have also been shown to influence receptor function (please see below for pathogenicity).

Anti-Caspr2 antibody epitope was mapped in 2015 [37, 38]. Caspr2 is an axonal protein located at the juxtaparanodes of myelinated axons and, along with Lgi1, is part of the VGKC complex. Caspr2 is made up of eight distinctive subdomains. From the N- terminus, these include the following: discoidin (Disc), lamininG (Lam1), lamininG (Lam2), epidermal growth factor 1 (Egf1), fibrinogenC (FibC), lamininG (Lam3), Egf (Egf2), and lamininG (Lam4) (Fig. 1d). The specificity of anti-Caspr2 antibody was also investigated using a series of deletion constructs, and then antibody-positive patient sera were tested by cell-based assay followed by immunocytochemistry [37] or flow cytometry [38]. A variety of different constructs were created involving single and multiple domain deletions to uncover the subdomain(s) involved in antibody binding. Autoantibody immunoreactivity was found to be specific to the extracellular N-terminal domain of Caspr2 and that autoantibodies target multiple subdomains, with the Disc domain containing the major target epitope. Indeed, the ΔDisc single domain deletion construct was the only one that showed significantly reduced immunoreactivity, but without a complete abrogation of binding [37]. Therefore, Olsen et al. concluded that while a major epitope is contained within the Disc domain of Caspr2, additional unknown epitopes also exist [37, 38]. The second study supports these conclusions [38] and also showed that N-terminal Disc and Lam1 domains are major antigenic targets in sera and CSF [38] (Fig. 1d). Furthermore, deglycosylation by tunicamycin or Peptide-N-Glycosidase F (PNGase F) did not prevent anti-Caspr2 antibody recognition, suggesting that N-linked glycosylation sites on Caspr2 are not important for antibody binding [37].

Anti-GAD65 antibodies have been associated with various syndromes including encephalitis, stiff person syndrome (SPS), cerebellar ataxia, epilepsy, and type 1 diabetes [25, 39–42]. GAD65 belongs to the pyridoxal 5′-phosphate (PLP)-dependent enzymes, involved in the synthesis of the neurotransmitter GABA [43, 44]. GAD65 consists of a PLP-binding domain, C-terminal domain (CTD), catalytic loop (CL), and N-terminal domain (NTD). In type 1 diabetes, the major target of anti-GAD65 antibodies have been mapped to PLP- and C-terminal regions [45, 46]. However, using competition binding with recombinant monoclonal antibodies, anti-GAD65 antibodies from stiff person syndrome were found to recognize a linear epitope on residues 4–22 and two conformational epitopes spanning residues 308–365 and 451–585 in ~30 and ~70 % patients, respectively [47] (Fig. 1e). These antigenic targets were not recognized by serum antibodies from patients with type 1 diabetes [47]. This suggests that epitopes could be different between diseases. Furthermore, differences in autoantibody epitope may also explain variations between patient clinical phenotype even though they are all anti-GAD65 antibody-positive. A more recent study aimed to examine epitope specificity among all anti-GAD65 antibody-positive patients with different neurological syndromes [48]. They found that patients predominantly recognized the middle domain, corresponding to the catalytic region of GAD65 (aa221-444) (Fig. 1e). N- and C-terminals were also recognized, but to a lesser extent, and there was no GAD-specific epitope that defined any neurological disorder [48].

## Autoimmune demyelinating diseases

Patients with demyelinating diseases, including acute disseminated encephalomyelitis (ADEM), neuromyelitis optica spectrum disorder (NMOSD), bilateral optic neuritis (BON), and chronic relapsing inflammatory optic neuropathy (CRION), have been found to be associated with antibodies against conformationally intact MOG [21, 49–55] (recently reviewed in [51]). MOG is a glycoprotein from the immunoglobulin superfamily, localized to the outermost layer of myelin (Fig. 1f), allowing easy access for potential pathogenic autoantibody binding. To gain precise insight into anti-MOG antibody recognition, 111 patient sera derived from MS, NMOSD, ADEM, or CRION patients were analyzed by live cell-based assay and flow cytometry after binding on MOG mutated by point mutagenesis within its extracellular immunoglobulin (Ig)-like domain [56]. Overall, seven epitopes were distinguished, and all were located within the ß strands of MOG, with the most frequently recognized epitope positioned in the CC’-loop within the extracellular Ig-like domain. Interestingly, the major epitope was a proline at position 42 within the CC’-loop, and the second most commonly recognized epitope was at positions 103 and 104 within the FC’-loop (Fig. 1f). The epitope specificity was similar in all of the disorders analyzed. Interestingly, position 42 is occupied by a serine in mouse MOG (mMOG). Due to mutation at P42, patient sera that recognized human MOG did not recognize mMOG, suggesting that the use of a mouse model to study anti-MOG antibody pathogenicity may be difficult due to failure of human autoantibody to recognize mMOG.

Arguably, the most recognized autoantibodies in demyelination are anti-AQP4 antibodies that are associated with neuromyelitis optica (NMO), a severe demyelinating disorder affecting the optic nerve and spinal cord. Anti-AQP4 antibody-positive patients present with longitudinal extended transverse myelitis and recurrent optic neuritis [57, 58]. Serum anti-AQP4 antibodies are polyclonal and have been shown to bind to conformational epitopes and linear peptides [59]. Epitope mapping has been complicated by the tendency of AQP4 to multimerize into membrane-bound homo- or heterotetramers and higher order structures called orthogonal arrays of particles (OAPs) [60]. A study screened for anti-peptide antibodies by ELISA and found that anti-AQP4 antibody-positive sera were reactive against three different peptides of AQP4, including aa1-22 (42.9 % of patients), aa88-113 (33 %), and aa252-275 (23.8 %). All epitopes identified were found to localize to the intracellular domains of the isoform M23 of AQP4 (AQP4-M23) [61] (Fig. 1g). However, when conformational APQ4-M23 was used, other epitopes were determined (Fig. 1g), and it is likely that these drive disease-relevance and pathogenicity. Two main conformational epitopes were found in extracellular loop A (aa61-64) and loop C (aa146-150) [62] (Fig. 1g). Mutations at W227 in the third extracellular loop E also influenced autoantibody binding in experiments using double loop A/E and C/E mutants. The importance of the conformation was also confirmed by a further study in which a point deletion at D69 within the second transmembrane domain altered the structural rearrangement of extracellular loop A and impaired antibody binding in 85.7 % of NMO patients [63]. These studies emphasize once again how the role of the conformation into epitope assembly can be teased out by the choice of methodology. Interestingly, another recent study focused on the epitope bound by recombinant anti-AQP4 antibodies (rAb) derived from CSF plasmablasts of NMO patients and had the added advantage of dealing with monoclonal rAb rather than patient polyclonal serum. They found that the amino acids within loop C (T137/P138, Val150, and N153) and loop E (H230/W231) were important for antibody binding (Fig. 1f), as well as an influence of loop A on rAb binding [64]. These epitopes are also bound by human serum autoantibodies as it has recently been confirmed by cell-based assay [65].

## Neuropsychiatric SLE

**Fig. 2:**
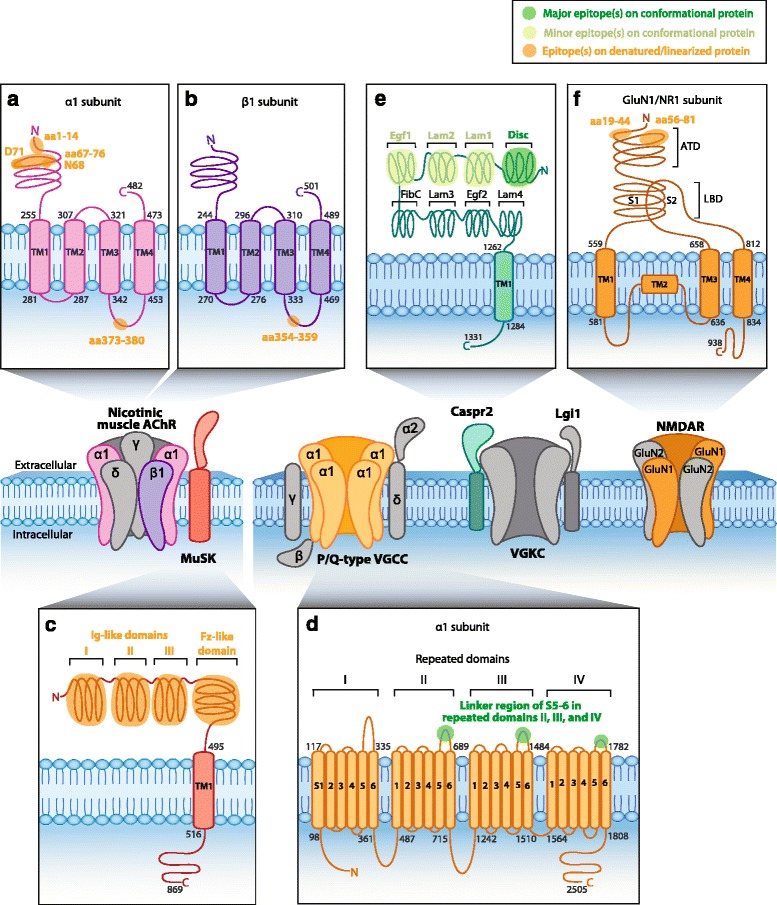
Epitopes of human PNS autoantibodies. Nicotinic muscle acetylcholine receptor (AChR) and muscle-specific kinase (MuSK, O15146) are postsynaptic muscle proteins. Anti-AChR antibodies target an extracellular epitope on the α1 subunit within amino acids (aa)1-14 [79] and aa67-76 [33]. Cytoplasmic epitopes have also been mapped in the α1 subunit at aa373-380 (**a**, P02708) and aa354–359 in the β1 subunit (**b**, P11230) [82]. **c** MuSK contains three immunoglobulin (Ig)-like domains and a Frizzled (Fz)-like domain. Anti-MuSK antibodies bind to the three N-linked Ig-like domains and the Fz-like domain [83–85]. **d** The neuronal P/Q-type voltage-gated calcium channel (VGCC) consists of four repeated domains, each containing six segments (S), with a linker region between S5 and S6. Anti-P/Q-type VGCC antibodies recognize epitopes in the linker region of repeating domains II–IV [94–96]. **e** Neuronal anti-contactin-associated protein 2 (Caspr2, Q9UHC6) antibodies recognize the extracellular N-terminal half (domains I–IV) but most commonly bind to an epitope within the discoidin (*Disc*) domain [37, 38]. **f** Neuronal *N*-methyl-D-aspartate receptor (NMDAR) contains an extracellular amino terminal domain (*ATD*) and a ligand-binding domain (*LBD*) formed by segment 1 (*S1*) and segment 2 (*S2*). Anti-NMDAR antibodies recognize aa19-44 and aa56-81 in the ATD of the GluN1 subunit (**f**, Q05586) [72]. Human protein topology, i.e., aa sequences and transmembrane domains (TM), is adapted from UniProt database, and UniProt identifiers are shown between *brackets*. Diagrams do not depict protein crystal structure. *Green* highlights major (*dark green*) and minor (*light green*) epitopes mapped by methods which retain the native in vivo conformational structure of the protein, such as cell-based assays. *Orange* highlights epitopes determined by methods which denature protein, such as western blots and ELISAs (Table 1)

Neuropsychiatric symptoms such as cognitive dysfunction, mood disorders, and/or psychosis develop in 17–75 % of systemic lupus erythematosus (SLE) patients [66], manifesting as neuropsychiatric systemic lupus erythematosus (NPSLE). A broad range of autoantibodies has been implicated in NPSLE, including those directed against native DNA, ribosomal P proteins, phospholipids, a proliferation-inducing ligand, and histones. The hallmark anti-DNA antibodies in SLE have generated substantial interest in the context of neuropsychiatric syndromes due to their cross-reactivity with the GluN2 (NR2) subunits of NMDAR. This connection was uncovered by screening a phage display peptide library with the R4A antibody which targets murine double-stranded DNA. From the phage library, a five amino acid consensus sequence, DWEYS, was identified on the GluN2a (NR2a) and GluN2b (NR2b) subunit of NMDAR in residues 283–287 [67] (Fig. 1b). Indeed, screening of serum and CSF identified a proportion of SLE patients that harbor anti-GluN2 (NR2) antibodies [67–70]. While several attempts have been made to correlate anti-GluN2 antibodies with clinical symptoms in patients, the data are inconsistent. Interestingly, anti-NR2 antibodies have also been detected in Rasmussen’s encephalitis and chronic progressive epilepsia using western blot [71]. Recently, serum and CSF from patients with focal NPSLE and peripheral involvement were tested against peptide sequences of GluN1 (NR1) subunit of NMDAR [72]. Antibodies recognized an epitope within the N-terminal extracellular domain at aa19-44 and aa56-81 using ELISA (Fig. 2f). However, the relevance of anti-GluN1 antibodies to NPSLE pathogenicity and their conformational epitopes have yet to be determined.

## Autoimmune neuromuscular junction disorders

Antibody-mediated diseases have also been associated with the PNS. Identification of the main autoantigens involved in PNS disorders has preceded many of the autoantigens in CNS disorders, possibly due to the greater accessibility to the PNS or to the now-refuted dogma that the brain was impenetrable to antibodies. However, a number of new targets have been identified for a number of disorders, highlighting ongoing development of autoimmune neurology.

### Myasthenia gravis

Myasthenia gravis (MG) is a peripheral autoimmune neuromuscular junction (NMJ) disorder, one of the earliest discovered naturally occurring autoimmune disorders mediated by antibodies. In 1973, antibodies against nicotinic muscle-type AChR were discovered in patient sera and in an experimental model of MG [73, 74]. Anti-AChR antibodies are responsible for pathophysiology in approximately 85 % of MG patients [28, 75], disrupting synaptic differentiation to cause muscle fatigue and weakness. The remainder of patients mainly harbors anti-MuSK antibodies (~10 %) or antibodies against a more recently identified antigenic target, Lrp4 (~5 %). While anti-AChR and anti-Lrp4 antibodies are mutually exclusive in MG, double seropositive cases occur for both anti-AChR or anti-Lrp4 and anti-MuSK antibodies [76].

Fundamentally, the nicotinic muscle-type AChR is a pentameric transmembrane glycoprotein of stoichiometry α_2_βγδ, in which the subunits are arranged around a central ion pore (Fig. 2). In adult muscle, a ε subunit occurs in place of the fetal γ subunit [77]. B cell epitopes have been identified on all five AChR subunits of fetal and adult forms, and the main immunogenic region is located on the acetylcholine-binding α1 subunit [78]. Early studies mapping the binding sites of anti-AChR antibodies looked at binding between synthetic peptides and rat monoclonal antibodies which recognized AChR from various species, including humans. The main immunogenic region of AChR was localized to aa67-76 on the extracellular loop of the α1 subunit using a solid-phase radioimmunoassay where 14-20mers were incubated with the different monoclonal antibodies [33]. The importance of the α1 subunit was then confirmed by competition binding radioimmunoassay examining the adsorption of these monoclonal antibodies by the chosen peptide. Results of a later study indicated that interaction of the main immunogenic loop with aa1-14 on the N-terminal α-helix produces a conformational epitope on AChR that significantly augments the degree of antibody binding [79]. The amino acids at positions 68 and 71 appeared to be crucial contact sites, as substitutions of these residues with L-alanine nearly abolished monoclonal antibody binding [80, 81] (Fig. 2a). Autoantibodies against intracellular portions of AChRs have also been detected in MG patients, targeting a variety of subunits [82]. Cytoplasmic epitopes reported thus far include aa373-380 of the α1 subunit (Fig. 2a) and aa354-359 of the β1 subunit [82] (Fig. 2b).

The extracellular domain of MuSK, which comprises three N-terminal Ig-like domains and a frizzled (Fz)-like domain (Fig. 2c), has also demonstrated antibody immunoreactivity in MG. A major epitope appears to be contained within the first Ig-like domain, as most anti-MuSK sera bind to this domain by ELISA [34, 83]. This result was also shown by earlier studies using radioimmunoassays in which most anti-MuSK patient sera recognized constructs comprising the first two extracellular Ig-like domains of MuSK [84–86]. A proportion of sera also bound to a construct of the combined third Ig-like and Fz-like domains [84] (Fig. 2c). Immunoreactivity to Fz-like domain was also confirmed by ELISA [34]. Further studies are necessary to dissect the binding specificity within each of these regions, particularly the highly immunogenic first Ig-like domain. Evidence from in vitro solid-phase assays suggests that antibodies against Lrp4 disrupt the association between Lrp4 and its ligand, agrin, to impair neuromuscular transmission [30]. It has been speculated that epitopes may be located within two specific LDLa domains in the extracellular region of Lrp4, since these sites normally appear to enhance Lrp4-agrin interaction [76]. However, epitope mapping studies have not yet examined this possibility nor investigated alternative antigenic sites.

Autoantibodies against titin, a large intracellular protein of striated muscle, have been detected and well-studied in MG patients, mainly those who are positive for anti-AChR antibody with ~40 % of MG patients positive for both antibodies [87]. Double positivity between anti-MuSK and anti-titin, and anti-Lrp4 and anti-titin antibodies has been detected in 15 % of patients [87]. Stergiou et al. have shown that 13 % of triple seronegative MG patients (without anti-AChR, anti-MusK, and anti-Lrp4 antibodies) had anti-titin antibodies [87]. The merit of anti-titin antibody as predictor of disease severity or thymoma in MG has been demonstrated by several investigators [88, 89]. An early study screened a human heart cDNA expression library with serum from five MG patients and mapped epitopes to Ig C2-like and fibronectin type III-like motifs [88]. This 30-kDa segment of titin, called MGT30, is considered the main immunogenic region of titin. MG thymoma patient sera reacted strongly with MGT30-expressing *E. coli* [87].

### Lambert-Eaton myasthenic syndrome

LEMS has also been shown to cause disruptions at the NMJ, leading to symptoms like proximal muscle weakness and autonomic dysfunction [90]. Eighty-five to ninety percent of LEMS cases are associated with autoantibodies against presynaptic VGCC [91–93], which impair neuromuscular transmission by diminishing the release of acetylcholine. VGCCs consist of four α1 subunits, which form the voltage sensor pore, and accessory subunits β, α2/δ, and γ (Fig. 2). Autoantibodies in LEMS patients mainly target the α1 subunit of P/Q-type VGCC. The VGCC α1 subunit consists of four repeated domains (I–IV), each with six transmembrane segments (called S1-S6) joined by linker regions. A radioimmunoassay in which synthetic peptides of the linker region between S5 and S6 from the four repeated domains were incubated with LEMS patient sera detected binding to the linker region from domains II and IV [94, 95]. An additional study identified a possible conformational epitope within the linker region between S5 and S6 of domain III [96] (Fig. 2d). Antibodies against the auxiliary β subunit, which is anchored to a cytoplasmic region of the α1 subunit of VGCC, have also been detected in a substantial proportion of LEMS patients [97–99].

An additional epitope target in LEMS is the synaptic vesicle protein synaptotagmin I [100]. Anti-synaptotagmin I antibody has been detected in a small proportion of patients, including several that were anti-VGCC antibody negative [93]. Synaptotagmin I localizes to the synaptic vesicle membrane where it functions as a calcium sensor to regulate neurotransmitter release. Despite synaptotagmin membrane location, portions of its N-terminus are likely to be exposed during exocytosis [101], enhancing its accessibility to circulating antibodies.

### Acquired neuromyotonia

Acquired neuromyotonia (NMT), unlike the myasthenic syndromes discussed above, involves an aberrant increase in acetylcholine activity at the NMJ, causing peripheral nerve hyperexcitability. Early studies implicated antibodies against VGKC, which were detected in 40 % of NMT sera [102]. Following this, immunoprecipitation studies using ^125^I-α-dendrotoxin-labeled VGKCs revealed specificity of NMT antibodies towards three Shaker-like (K_V_1) VGKC α subunits, K_V_1.1, K_V_1.2, and K_V_1.6 [103, 104]. Because patient sera were shown to bind to recombinant forms of the same three K_V_1 channel proteins expressed on transfected Xenopus oocytes, it was suggested that a conformational unmapped epitope was located on these subunits [104]. However, recent reevaluation of autoantibody specificity against VGKC by cell-based assays has demonstrated that bound antigens were in fact mostly Caspr2 and sometimes Lgi1 with only a minority of patients binding to K_v_1 VGKC [13, 15]. As previously discussed, anti-Caspr2 antibody has been shown to target the extracellular N-terminal domain of Caspr2 (Fig. 2e) [37]. Moreover, findings of anti-VGKC complex antibody-positive NMT patients in which epitope targets are unaccounted for may suggest involvement of other VGKC subunits or unidentified components of the VGKC complex [105].

### Autoimmune neuropathies

**Fig. 3:**
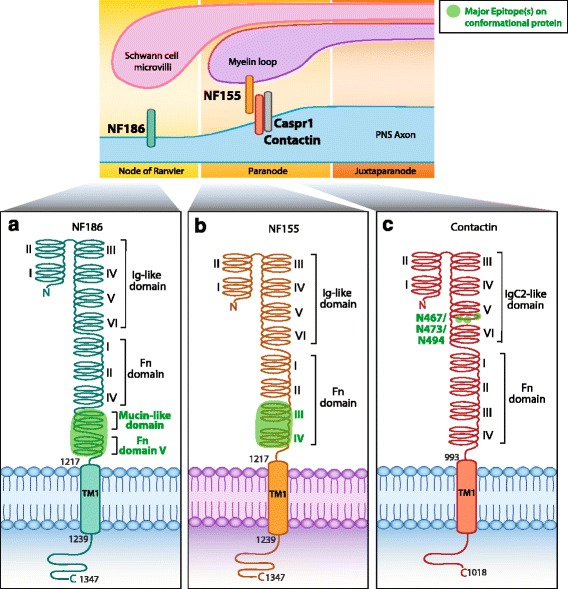
Epitopes of human PNS autoantibodies at the node of Ranvier. There are two isoforms of neurofascin (NF). **a** The neuronal isoform NF 186 (*NF186*, O94856) that resides within the node. **b** The glial isoform NF 155 (*NF155*, O94856) that resides on the myelin loop within the paranode. Anti-NF186 antibodies target the extracellular fibronectin type III (Fn) V (or Fn5) and Mucin-like domain. Anti-NF155 antibodies target extracellular domains Fn III (or Fn3) and Fn IV (Fn4) [107]. **c** Antibodies directed against neuronal contactin (Q12860) target N-glycosylation sites N467, N473, and N494, within the extracellular immunoglobulin (Ig) C2-like domain 5 [108]. Human protein topology, i.e., aa sequences and transmembrane domains (TM), is adapted from UniProt database, and UniProt identifiers are shown between *brackets*. Diagrams do not depict protein crystal structure. *Dark green* highlights major epitopes mapped by methods which retain the native in vivo conformational structure of the protein, such as cell-based assays (Table 1)

The node of Ranvier and the adjacent paranode of myelinated axons contain important cell adhesion molecules including neurofascin and contactin. Contactin interacts with oligodendrocyte-specific neurofascin-155 (NF155) at the paranode via N-glycans, whereas neuron-specific neurofascin-186 (NF186) is localized at the node. The extracellular domains of NF155 and NF186 were observed to be targets of autoantibodies in MS patients, and these autoantibodies have been shown to exacerbate disease in experimental acute encephalomyelitis (EAE) mouse model [26]. Furthermore, anti-neurofascin antibodies have been detected in autoimmune PNS neuropathies, such as chronic inflammatory demyelinating polyneuropathy (CIDP), Guillain-Barre syndrome (GBS), and its variants: acute motor axonal neuropathy (AMAN) and acute inflammatory demyelinating polyradiculoneuropathy (AIPD) [106, 107]. The target epitopes of anti-NF155 and anti-NF186 antibodies in CIDP patients were mapped by cell-based assay to fibronectin type III (Fn) domains Fn3 and Fn4 (Fig. 3a) and Fn5 and Mucin-like domain (Fig. 3b), respectively [107]. Interestingly, CIDP and GBS patients also harbor anti-contactin 1 antibodies [27, 108]. Anti-contactin 1 antibody binding was highly dependent on N-glycosylation in a case series of four CIDP patients, and one patient more specifically targeted N-glycosylated asparagines at positions 467, 473, and 494 [108] (Fig. 3c).

Approximately 60 % of GBS patients also harbor anti-ganglioside antibodies [109]. Depending on the GBS subtype, these autoantibodies may target the myelin sheath and Schwann cells, or the axons of peripheral nerves [110], and might be pathogenic [111, 112]. Gangliosides are a family of sialic acid-containing glycosphingolipids and constitute a viable target for circulating autoantibodies due to their location in the outer leaflet of the cell membrane. IgG antibodies against the ganglioside GM1 were first detected in sera of GBS patients by ELISA [113]. Although anti-GM1 antibodies from the IgG isotype are indeed the most frequently reported in the pure motor variant of GBS [114], autoantibodies targeting different gangliosides appear to correlate with distinct clinical phenotypes [110]. For instance, another form of GBS called Miller-Fisher syndrome is characterized by antibodies against ganglioside GQ1b [115]. More recently, it has been shown that autoantibodies in a small number of GBS patients recognize complexes formed by two different gangliosides, for instance GD1a/GD1b and GD1a/GM1 [116, 117]. This knowledge has improved anti-ganglioside antibody detection rates and led to reclassification of a small number of patients who were seronegative for autoantibodies against individual gangliosides [116].

Antibodies against nicotinic ganglionic AChR are strongly associated with patients with autoimmune autonomic ganglionopathy (AAG), although they are found in some patients with other autonomic neuropathies [118]. An early study by Vernino et al. found that approximately 50 % of patients with acute or subacute forms of AAG harbor antibodies against ganglionic AChR [119]. However, a later study showed that the clinical associations of autoantibodies against ganglionic AChR may be broader than previously thought, with many patients harboring diverse neurological presentations including peripheral neuropathies, dysautonomia, and encephalopathy. Cancers were also found in one third of the examined cohort [120]. Ganglionic AChR can be distinguished from muscle AChR by the presence of two α3 instead of α1 subunits, and increased expression in the peripheral autonomic ganglia, where it is necessary for fast synaptic transmission. While it appears that all seropositive AAG patients have anti-ganglionic AChR antibodies that target the α3 subunit [121], a minor proportion of patients also harbor additional immunoreactivity against the AChR β4 subunit [122] that commonly associates with α3. Correlation between ganglionic anti-AChR antibody titers and disease severity and decrease with treatment and clinical improvement [119], as well as animal studies [123, 124], have implied that these antibodies have pathogenic potential. Specific epitopes on gangliosides and ganglionic AChR have not been mapped.

## Relevance of identifying the epitopes of autoantibodies

Currently, the mechanism by which an autoimmune response is initiated is unclear, and where exactly CNS autoantibodies are generated has been a subject of much interest. Studies have shown that disease-associated B cells are found both within the periphery and CNS and that the cells are clonally related, for example in MS [125]. Palanichamy et al. demonstrated that an exchange of immunologically active clusters of related B cells occurs between the CNS and peripheral blood compartments. Also, the majority of B cell maturation occurs outside the CNS [126]. These recent discoveries highlight the relevance of using patient sera for autoantibody detection and epitope analysis studies, since B cell maturation can occur in the periphery, specifically in the cervical lymph nodes [126].

While mapping of epitope will likely help us understand autoantibody function, conversely it may also be true that an understanding of antibody role, which could influence antigenic function, will help to predict or uncover the epitopes. Overall, knowledge of the autoantibody epitope target may provide valuable insight into the earliest immune mechanisms of autoimmunity, lead to improved diagnosis, allow more precise testing, and enable development of epitope-specific therapies. In addition, determining whether the target epitope is conserved between species, for example, between human and rodent, may have relevance to pathogenic studies in animal models.

## Epitope and antibody detection

Some of the autoantigens detected in the antibody-mediated disorders described above can have two or more isoforms. Knowledge of autoantibody isoform specificity may be important for diagnosis and will ensure precise testing. For example, AQP4 has two isoforms, AQP4-M1 and AQP4-M23. Inconsistent diagnostic results have emerged due to the isoform selected for anti-AQP4 antibody testing [127–129]. Untagged AQP4-M23 isoform used in a live cell-based assay was the most ideal method, yielding a specificity of 100 % and sensitivity of 74.4 % [130]. Another study found that AQP4-M1 cell-based fluorescence assay was the most reliable method for antibody testing and serological diagnosis [131], and another large study did not find significant differences between AQP4-M1 and AQP4-M23 [132]. However, recently, a multi-center study further confirmed the value of using AQP4-M23 isoform for optimal sensitivity [133].

## Epitope and antibody pathogenesis

Autoantibodies targeting extracellular, rather than intracellular, domains of an antigen have a higher probability of being pathogenic by modulating receptor function, which can be studied in vitro and in vivo. However, since epitopes may vary between species, matching epitope targets between human autoantibodies and murine models is important for animal studies. For instance, the majority of patients with anti-MOG antibodies did not recognize conformational intact mMOG [56], whereas epitopes recognized by anti-NMDAR antibodies are similar between the two species [35], or at least share some cross-reactivity as in the case of anti-AQP4 antibodies [62, 134]. Longitudinal studies of autoimmune neurological disorders in humans are necessary to substantiate findings from animal models and determine whether the same mechanisms are relevant to human disease. Based on these results, decisions can be made as to whether the therapies that have proved effective in animal models are translatable to human disorders.

Understanding the antibody epitope will enable inquiry into mechanisms of antibody pathogenic potential. In the case of NMDAR, residues N368/G369 are located within the bottom lobe of ATD, in close proximity to the hinge of the two ATD lobes that are important for receptor physiology. Outside-out single channel recordings were measured, which showed that anti-NMDAR antibody binding prolongs the open time of the receptor. These results suggest that antibodies influence channel function, and knowing where antibodies bound also helped to hypothesize on how they can hinder their target function. Previous studies support anti-NMDAR antibody pathogenicity as they can induce receptor internalization [135, 136] and alter receptor trafficking [137]. Recently, two studies have endeavored to assess anti-NMDAR antibody pathogenicity in animal models [138, 139]. Planaguma et al. used dialyzed patient and control CSF samples before cerebroventricular infusion using osmotic pumps. Upon injection of patient samples, but not control samples, mice developed progressive memory deficits and depressive-like behaviors over the next 18 days but did not have other affected behavioral or locomotor activities [138]. Wright et al. investigated the epileptogenic potential of anti-NMDAR antibodies. C57BL/6 mice were also injected intracerebroventricularly, but with purified IgG from CSF of anti-NMDAR antibody-positive patients or healthy controls. Anti-NMDAR IgG antibodies alone did not produce spontaneous seizures. However, when mice were challenged intraperitoneally with chemo-convulsant pentylenetetrazol, mice injected with anti-NMDAR antibodies had more convulsive seizures than healthy controls within 2 days [139]. Although these studies are complementary and commendable, the animal models did not entirely recapitulate human disease phenotype. For example, the movement disorders observed in most anti-NMDAR encephalitis patients were not present in either models, and this illustrates the ambiguity and difficulty in assessing antibody pathogenic mechanisms in vivo.

To evaluate the functional effect of anti-AMPAR antibodies, purified anti-AMPAR IgG antibodies from patients or controls were incubated on primary rat cerebrocortical neurons, and miniature excitatory postsynaptic current (mEPSCs) recordings were measured. Compared to control IgG, it was found that anti-AMPAR antibodies altered mEPSCs in cultured neurons [32]. Therefore, binding of anti-AMPAR antibodies to the bottom lobe of ATD may alter the electrophysiology of AMPAR and affect normal function of neuronal receptor upon binding.

Different amino acid recognition can lead to epitope-specific pathogenic effects as in the case of anti-GAD65 antibody, for which pathogenic evidence is mounting [47, 140, 141]. Indeed, antibodies directed to residues 308–365 induce intense and rapidly weaning effects on motor and cognitive functions, and antibodies to residues 451–585 induce sustained medium-sized effects in cerebellum-injected rats [142]. These results have to be discussed in the context of the intracellular location of GAD65, and although the antibody access has not been fully explained yet, the uptake of immunoglobulins into neurons could occur via antigen-dependent or antigen-independent mechanisms [142, 143].

Due to their IgG isotype, some antibodies have the capacity to activate immunological cytotoxic mechanisms. This has been the case for anti-Caspr2 and anti-MOG antibodies in encephalitis and demyelinating disorders, respectively. Indeed, both human anti-Caspr2 and anti-MOG antibodies have been shown to activate complement in vitro [50, 144]. However, evidence of anti-MOG antibody pathogenicity in demyelinating disorders is still limited. In vivo and in vitro animal studies indicated that anti-MOG antibodies mediate complement-mediated lysis [145, 146]. One study suggested a mechanism of cell-mediated cytotoxicity, as sera from pediatric samples were found to induce natural killer cell activation to surface-expressing MOG [21]. Using MOG-transfected HEK293-A cells, another study demonstrated that anti-MOG antibodies, at high titers, are able to activate complement to cause lysis of MOG-expressing cells, whereas antibodies at low titers did not [50]. A more recent study observed loss of cytoskeletal organization in MOG-expressing MO3.13 cells incubated with demyelinating pediatric purified IgG, which contained anti-MOG antibodies [147]. Although the above studies have started to uncover the effector mechanisms of anti-MOG antibodies, how exactly autoantibody binding to its epitope contributes to these mechanisms is yet to be established.

Antibody pathogenicity has also been demonstrated in PNS antibody-mediated disorders. For instance, pathogenicity of anti-AChR antibodies is evidenced by the passive transfer of experimental autoimmune myasthenia gravis (EAMG) following anti-AChR monoclonal antibody injection. Antibodies targeting the AChR main immunogenic region appear to be particularly potent, with one study showing that several different monoclonal antibodies targeting this region of AChR efficiently induced myasthenic symptoms in rats [148]. The monoclonal antibodies used in this study were rat IgG1 or IgG2 against AChR from fish electric organ or mammalian muscle, and each antibody caused an approximate 50 % reduction in AChR quantal content. In contrast, antibodies directed against intracellular portions of the α1 subunit or δ subunit, and monoclonal antibodies against the extracellular portion of the β1 subunit of AChR did not cause EAMG. Further to this, the effects of monoclonal antibodies against the main immunogenic region were not as potent as anti-AChR sera, suggesting that other serum factors play a role in enhancing myasthenic symptoms. These factors may be other IgG subclasses or polyclonal antibodies that target other regions of AChR. As mentioned above, anti-MuSK antibodies target the Ig-like domain 1. Interestingly, that region contains residue I96 that is essential for interaction between MuSK and Lrp4 [149]. Anti-MuSK antibodies could be pathogenic via the prevention of MuSK-Lrp4 binding, either by hindering binding or by modifying MuSK topology. Passive transfer of MuSK MG patient sera or IgG [84, 150, 151] and the extracellular domain of Lrp4 [152] have demonstrated similar pathogenic effects in mice. In the context of LEMS, injection of a peptide sequence of synaptotagmin containing residues 20–53 stimulated antibody production in rats and caused electrophysiological defects at the NMJ, including reduced acetylcholine release [153]. Similarly, animal transfer studies involving autoantibodies against *Shaker*-like VGKC [102] cause aberrations in synaptic transmission. There is further evidence for the autoimmune etiology of NMT in that it may be associated with myasthenic syndromes and SLE.

Purified SLE anti-DNA antibodies following injection into mouse brain also have neurotoxic effects. This effect might occur via their binding to NMDAR as GluN2 blocker MK-801-treated mice had no neuronal injury after antibody injection [67]. Conversely, not all antibodies show pathogenic features, as seen in LEMS. Antibodies are raised against the VGCC β subunit in some patients but are unlikely to be a pathogenic factor in LEMS as rats immunized with recombinant β subunit fail to develop neuromuscular defects, despite exhibiting high antibody titers [99].

### Epitope and potential therapy

Uncovering the autoantibody epitope target can aid in the development of novel therapies tailored to patient needs including epitope-specific therapies, such as decoy antigen therapy. Decoy antigen therapy is the use of a soluble epitope to block antibody binding to target brain antigen [154, 155]. In the case of EAMG, a therapeutic vaccine consisting of bacterially expressed cytoplasmic domains of human AChR subunits was found to reduce the development of chronic EAMG in rats. The authors found that the mechanism behind the novel therapy was to change the isotype of the antibody response from IgG2b to IgG1. Furthermore, they show that injection of AChR cytoplasmic domains in adjuvant is promising as a safe, effective, antigen-specific, rapidly acting, and long-lasting approach to therapy of MG [156]. Other additional therapeutic strategies attempt to prevent antibody-dependent pathogenicity by a “blocker therapy”. Anti-AQP4 rAbs with a mutated Fc region to hinder antibody functionality were utilized in a competition assay with anti-AQP4 antibodies from NMO patient sera. rAbs were capable of blocking AQP4 binding by pathogenic patient antibodies, thereby impeding downstream antibody-mediated complement and cell cytotoxicity in vivo and in vitro [157, 158]. Uncovering the autoantibody epitope can also aid to understand therapy sensitivity. Indeed, it could be hypothesized that specific epitope patterns could be correlated to therapy responsiveness, and therefore influence patient treatment.

## Potential triggers of antibody-mediated autoimmunity

Currently, it is unclear what initiates autoimmunity. One proposed hypothesis is that ectopic expression of antigens, for example by tumors, would activate autoimmune responses. Ovarian teratoma, found in 50 % of anti-NMDAR encephalitis adult patients, have been shown to express NMDAR [10, 135, 159]. However, these tumors are seldom detected in all patients, especially younger ones, who represent an important group of patients. Another proposed hypothesis is molecular mimicry, a phenomenon whereby infectious microbes express antigens which share structural homology with self-antigens, so immune responses initiated towards microbes may result in a reaction against the body’s own antigens [160, 161]. One study reports the mechanism of cross-reactivity between AQP4 and human T-lymphotropic virus 1 (HTLV-1). After matching one intracellular AQP4 epitope (described in [61]) to a highly similar sequence in the human TAX1BP1 protein involved in HTLV-1 replication, the investigators detected anti-AQP4 antibodies in mice immunized with the TAX1BP1-derived peptide [162]. These results suggest that a latent HTLV-1 infection could lead to TAX1BP1 antigen presentation and the production of anti-AQP4 antibodies, perhaps through T cell-mediated mechanisms [162]. Further experiments are required to validate these hypotheses in NMO.

Additionally, several studies report a progression to anti-NMDAR encephalitis following herpes simplex virus encephalitis (HSVE). In some cases, patients were initially diagnosed with HSVE, but later progressed to develop anti-NMDAR encephalitis or anti-D2R antibody-associated movement and psychiatric disorders that tested positive for anti-NMDAR and anti-D2R antibodies [163–167]. Although these studies have identified a link between HSV and development of autoimmune disorders, whether or not molecular mimicry is involved is still unclear. High neuronal antigen levels could be released during damage to the brain parenchyma after infection by neurotropic herpes simplex virus (HSV), therefore contributing to an activation of an autoimmune response. Additionally, anti-NMDAR encephalitis has also been observed to follow a varicella zoster brain infection in one patient [168]. On the other hand, some autoantibodies, for example anti-D2R antibodies, have been detected in disorders, such as group A Streptococcus-associated Sydenham chorea, that are clearly induced by bacterial infection, and molecular mimicry has been suggested between bacterial antigens and D2R using a rAb isolated from a patient [169]. There is a longstanding hypothesis of molecular mimicry in GBS, as about one quarter of cases are preceded by infection with *Campylobacter jejuni* [170]. Evidence for this hypothesis arose from a study in rabbits, where animals immunized with C. *jejuni*-like lipopolysaccharides (LPS) developed antibodies against a panel of ganglioside antigens [171]. The same study found that serum IgG from these rabbits and GBS patients bound gangliosides at the nodes of Ranvier. Molecular mimicry between gangliosides on the surface of neurons and C. *jejuni* LPS has since been supported by mass spectrometry and serological studies. LPS of the *C. jejuni* strains that cause GBS harbor motifs that resemble GM1, GD1a, and GQ1b gangliosides [172, 173]. Antibodies against other combinations of gangliosides are thought to induce related disorders like Miller-Fisher syndrome [110]. However, the fact that not all GBS patients are reactive to *C. jejuni* suggests that molecular mimicry might not be the only mechanism at play. Overall, determination of possible molecular mimicry relies on a linear comparison between pathogen and autoantigen sequences, and multiple confounders, such as conformational non-linear epitopes and polyclonality, exist, making it a challenging concept to prove.

Additionally, patients with rheumatoid arthritis, MG, LEMS, Crohn’s disease, SLE, systemic vasculitis, and IgA nephropathy have all been shown to have altered immunoglobulin glycosylation [174–178]. It has been recently suggested that these N-glycans present within Fc region of the antibody molecule could influence antibody effector function and therefore play a role in autoimmunity, although this concept would need further validation [179].

## Potential mechanisms of double autoantibody positivity

Mapping of autoantibody epitopes has shown that patients, individually or in clusters, can have different recognition patterns. They can recognize either a single dominant epitope, a combination of major and minor epitopes, or multiple different epitopes of the same antigen. The presence of multiple epitopes may suggest that a polyclonal antibody response is active.

The studies discussed in this review have generally reported single autoantibody immunoreactivity associated with its respective disorder. For example, anti-NMDAR antibodies are detected in anti-NMDAR encephalitis. However, several recent studies have reported the presence of double autoantibody positivity in a small number of patients. The phenomenon of double antibody positivity remains an understudied subject and has been observed in both CNS and PNS antibody-mediated disorders. The presence of autoantibodies against multiple antigens, including anti-ganglionic AChR antibody, was first reported in patients with paraneoplastic disorders and may be a potential biomarker to predict cancer [180]. In 2010, a case report of a 56-year-old man with non-paraneoplastic limbic encephalitis was found to be associated with anti-NMDAR and anti-VGKC antibodies [181]. A subgroup of limbic encephalitis patients positive for anti-GABA_B_R antibody was also found to be positive to other antigens including thyroid peroxidase (TPO), GAD65, sex determining region Y-box 1 (SOX1), or N-type VGCC [16]. Anti-GABA_B_R antibodies have also been observed in anti-GAD65 antibody-positive patients with paraneoplastic syndromes [182]. Multiple antibody positivity associated with cancer has been reported in several studies [182, 183]. Petit-Pedrol et al. (2014) recently reported five cases of anti-GABA_A_R and anti-GAD65 antibodies co-occurring in CSF and serum [17], and a single case report of a female with encephalitis was found to harbor anti-GABA_A_R and anti-GAD65 antibodies in CSF and serum [184]. Other double antibody positivity combinations that have been identified are anti-NMDAR and anti-GAD antibodies [185], anti-NMDAR and anti-D2R [165, 186], anti-GABA_B_R and anti-Hu [187], and anti-Caspr2 and anti-Lgi1 in a patient with Morvan syndrome [188]. Recently, patients positive for anti-MOG and anti-NMDAR antibodies were reported [189], and later on, double antibody positivity for anti-NMDAR and anti-GlyR and anti-MOG and anti-GlyR antibodies were also shown in two patients [190]. One study even reported triple antibody positivity, including anti-GABA_B_R, anti-Hu, and anti-NMDAR antibodies [187].

The occurrence of MG in LEMS patients is a widely accepted phenomenon [191]. Although diagnosis of MG in LEMS individuals has mainly been based on clinical presentation, tests for serum autoantibodies against AChR and VGCC revealed a number of double seropositive cases [192–194]. A case of triple antibody seropositivity has also been identified with anti-AChR, anti-VGKC, and anti-MuSK antibodies in a patient with MG and Morvan’s syndrome which is commonly associated with VGKC [195]. In SLE, one third of patients have anti-NMDAR antibodies [196]. In addition, a cancer-positive LEMS patient double positive for anti-VGCC and anti-GABA_B_R antibodies has been reported [197]. The coexistence of multiple anti-ganglioside antibodies is not uncommon, for instance, anti-GM1 and anti-GM1b in GBS patients [198]. Additionally, examples of seropositivity exist for anti-ganglioside antibodies together with other antibodies. One study indirectly detected immunoreactivity against nicotinic AChR in GBS patient serum, with the finding that serum interfered with the postsynaptic transmission of nicotinic AChR [199]. Anti-VGKC antibodies have been found along with antibodies against ganglioside GQ1b in variants of GBS [200].

These cases of double or multi-antibody positivity are interesting, although mechanisms behind this poly-immunoreactivity have not been elucidated. Underlying tumors could drive the generation of additional antibody seropositivity [180, 197]. Another possibility is the concurrence of two single antibody-specific disorders in the same patient, which is more likely the case for double anti-NMDAR and anti-MOG antibody and anti-AChR and anti-AQP4 antibody positivity [166, 201]. Multi-antibody positivity may be explained by the concept known as epitope spreading, in which persistent recognition and activation to self-antigens lead to chronic immune system activation [202]. Epitope spreading within the same antigen (intramolecular) or to different antigens (intermolecular) has predominantly been observed in animal models, particularly experimental autoimmune models of EAE and EAMG [203, 204]. Early reports of intermolecular epitope spreading date from the 1990s. Epitope spreading was reported in several mice models of MS, including (SJLxPL)F1, relapsing EAE (R-EAE), SJL/J, B10.PL, and Theiler’s murine encephalomyelitis virus-induced demyelinating disease (TMEV-IDD) [204–208]. The mechanism behind epitope spreading is unknown, but it has been suggested that local antigen presenting cells, such as dendritic cells, would initiate epitope spreading in CNS by activating naïve T cells [204]. EAMG can be induced by immunization of organisms with AChR, yielding humoral and cellular responses. When rats were immunized with a recombinant fragment of the extracellular AChR α subunit to induce EAMG, antibodies directed against a hidden intracellular loop of the subunit were detected in two of the immunized animals several weeks after an initial response to the extracellular portion [203]. The fact that the presence of antibodies against the intracellular epitope corresponded to increased disease severity in these animals suggests that epitope specificity may be an important determinant of disease outcomes.

In humans, epitope spreading has also been observed in pediatric MS [209]. Serum samples were collected in children with acquired CNS demyelinating syndromes (ADS) at time of initial attack, and at follow-up at 3 months when children were categorized to either have pediatric MS (ADS-MS) or a monophasic illness (ADS-mono). Immunoreactivity of patient serum samples was tested against a panel of 48 CNS antigens, including MOG, myelin basin protein (MBP), proteolipid protein, and CNPase. At initial attack, serum antibody pattern between ADS-MS and ADS-mono patients reacted to similar number of CNS antigens. After 3 months, ADS-MS patients reacted against a greater number of CNS antigens and bound to different epitopes within these antigens, while ADS-mono samples demonstrated a reduction in CNS antibody response [209]. This evidence is suggestive of intra- and intermolecular epitope spreading in patients with ADS-MS. Epitope spreading has also been observed in a third of anti-AQP4 antibody-positive patients (7/20) over a 15-year follow-up, whereas the majority (13/20) maintained a stable antibody binding pattern [65]. Additionally, in a 5-year longitudinal study on 11 patients harboring anti-MOG antibodies, there was constant epitope recognition with little evidence of intramolecular epitope spreading [56]. This was also observed in MG patients. A recent study examining anti-MuSK antibody-positive MG sera found that intramolecular epitope spreading was relatively uncommon within a minimum of 5 years of follow-up. However, when it occurred, immunoreactivity “spread” from the first and second Ig-like domains to the Fz-like domain [34].

Whether epitope spreading occurs over time and in which time frame is therefore important to determine. Such knowledge may prove useful in designing appropriate immunotherapies, like monoclonal antibodies or blocker peptides that bind specific epitopes at different stages of progression.

## Conclusions

As the field of antibody-mediated immune disorders continues to expand and as methodological practices improve, it is expected that the number of antibody-positive patients will rise and that new autoantigens will also be discovered. Numerous cases of patients who present with clinical features similar to antibody-positive patients have been reported, yet upon serological testing, they remain antibody-negative, suggesting the likelihood of unknown antigenic targets or other yet undescribed immunological pathways that could be T cell-driven. Our current understanding of antibody-mediated disorders and their antibody targets may assist in discovering not only other potential autoantigens but also their epitopes. For instance, NMDAR and AMPAR are ionotropic receptors with similar structure, and their target epitopes are located within the extracellular ATD. Another important ionotropic receptor is kainate receptor. The kainate receptors are a subtype of the ionotropic glutamate receptor. They are involved in synaptic transmission and contribute to postsynaptic excitatory currents in cortex, hippocampus, retina, and spinal cord. Extrapolating from the autoantigens already identified, we may be able to infer other potential autoantigens and their autoantibody epitope.

As mentioned, patients can harbor autoantibodies that target two different antigens, and some patient antibodies have been shown to recognize multiple epitopes of a single antigen. Mechanisms underlying these two occurrences remain unclear. Patients with double antibody positivity may be difficult to assess when it comes to diagnosis and treatment options. It is also unknown whether a polyclonal antibody response may be due to epitope spreading or ectopic antigen expression, and longitudinal analyses, as well as thorough clinical investigations, may help to investigate these phenomena. Future studies will also be needed to investigate whether the epitope specificity is similar between CSF and serum of patients. Greater knowledge of the epitope involved in autoantibody binding may ensure that appropriate therapies are administered and, in doing so, improve patient care.

## Abbreviations

Aa: Amino acids
AChR: Acetylcholine receptor
ADEM: Acute disseminated encephalomyelitis
ADS: Acquired demyelinating syndromes
AIPD: Acute inflammatory demyelinating polyradiculoneuropathy
AMAN: Acute motor axonal neuropathy
AMPAR: α-Amino-3-hydroxy-5-methyl-4-isoxazolepropionic acid receptor
AQP4: Aquaporin-4
ATD: Amino-terminal domains
BON: Bilateral optic neuritis
Caspr2: Contactin-associated protein-like 2
CIDP: Chronic inflammatory demyelinating polyneuropathy
CL: Catalytic loop
CNS: Central nervous system
CRION: Chronic relapsing inflammatory optic neuropathy
CSF: Cerebrospinal fluid
CTD: C-terminal domain
D2R: Dopamine-2 receptor
Disc: Discoidin
DNA: Deoxyribonucleic acid
DPPX: Dipeptidyl-peptidase-like protein-6
EAE: Experimental autoimmune encephalomyelitis
EAMG: Experimental autoimmune myasthenia gravis
ELISA: Enzyme-linked immunosorbent assay
FibC: FibrinogenC
Fz: Frizzled
GABA_A_R: γ-Aminobutyric acid receptor-A
GABA_B_R: γ-Aminobutyric acid receptor-B
GAD65: 65-kDa glutamic acid decarboxylase
GBS: Guillain-Barre syndrome
GlyR: Glycine receptor
HEK293: Human embryonic kidney 293 cells
HSV: Herpes simplex virus
HSVE: Herpes simplex virus encephalitis
ICC: Immunocytochemistry
Ig: Immunoglobulin
Lam: LamininG
LEMS: Lambert-Eaton myasthenic syndrome
Lgi1: Leucine-rich glioma-inactivated protein 1
Lrp4: Lipoprotein receptor-related protein 4
MBP: Myelin basic protein
mEPSCs: Miniature excitatory postsynaptic current
MG: Myasthenia gravis
mGluR5: Metabotropic glutamate receptor 5
mMOG: Mouse myelin oligodendrocyte glycoprotein
MOG: Myelin oligodendrocyte glycoprotein
MS: Multiple sclerosis
MuSK: Muscle-specific kinase
NF: Neurofascin
NMDAR: *N*-methyl-D-aspartate receptor
NMJ: Autoimmune neuromuscular junction
NMO: Neuromyelitis optica
NMOSD: Neuromyelitis optica spectrum disorder
NMT: Acquired neuromyotonia
NPSLE: Neuropsychiatric systematic lupus erythematosus
NTD: N-terminal domain
OAPs: Orthogonal arrays of particles
PLP: Pyridoxal 5′-phosphate
PNGase F: Peptide-N-Glycosidase F
PNS: Peripheral nervous system
rAb: Recombinant antibody
SLE: Systematic lupus erythematosus
SOX1: Sex determining region Y-box 1
SPS: Stiff person syndrome
TM: Transmembrane
TMEV-IDD: Theiler’s murine encephalomyelitis virus-induced demyelinating disease
TPO: Thyroid peroxidase
VGCC: Voltage-gated calcium channel
VGKC: Voltage-gated potassium channel.
